# Survey to measure the quality of life of patients with tuberculosis in Alexandria, Egypt: a cross-sectional study

**DOI:** 10.1186/s12913-023-09381-z

**Published:** 2023-05-24

**Authors:** Esraa Abdellatif Hammouda, Wahib Fayez Gobran, Reem Mohamed Tawfeek, Ola Fahmy Esmail, Rasha Ashmawy, Naglaa Youssef, Ramy Mohamed Ghazy

**Affiliations:** 1 Department of Clinical Research, El-Raml pediatric hospital, Ministry of Health and Population, Alexandria, Egypt; 2 Director of Chest Diseases, Ministry of Health and Population, Alexandria, Egypt; 3 Ministry of Health and Population, Alexandria, Egypt; 4 Egyptian Drug Authority, Cairo, Egypt; 5 Department of Clinical Research, Maamoura Chest Hospital, Ministry of Health and Population, Alexandria, Egypt; 6 Medical-Surgical Nursing Department, Faculty of Nursing, Cairo University, Cairo, Egypt; 7 Tropical Health Department, High Institute of Public Health, Alexandria University, Alexandria, Egypt; 8 Department of Medical-surgical Nursing, College of Nursing, Princess Nourah bint Abdulrahman University, Riyadh, Saudi Arabia

**Keywords:** Tuberculosis, Quality of life, WHOQOL-BREF

## Abstract

**Background:**

Assessment of quality of life (QoL) in patients with tuberculosis (TB) may improve healthcare providers’ understanding of the disease burden. This study aimed to investigate the QoL of patients with TB in Alexandria, Egypt.

**Methods:**

This cross-sectional study was conducted in chest clinics and main chest hospitals in Alexandria, Egypt. A structured interview questionnaire was used to collect data from participants through face-to-face interviews from November 20, 2021, until the June 30, 2022. We included all adult patients aged 18 years or above during the intensive or continuation phase of treatment. The World Health Organization (WHO) WHOQOL-BREF instrument was used to measure QoL, which includes the physical, psychological, social relationships, and environmental health domains. Using propensity score matching, a group of TB free population was recruited from the same setting and completed the questionnaire.

**Results:**

A total of 180 patients participated in the study: 74.4% were males, 54.4% were married, 60.0% were 18–40 years old, 83.3% lived in urban areas, 31.7% were illiterate, 69.5% reported insufficient income, and 10.0% had multidrug-resistant TB. The TB-free population group had higher QoL scores than the TB patients’ group: (65.0 ± 17.5 vs. 42.4 ± 17.8) for the physical domain, (59.2 ± 13.6 vs. 41.9 ± 15.1) for the psychological domain, (61.8 ± 19.9 vs. 50.3 ± 20.6) for the social domain, (56.3 ± 19.3 vs. 44.5 ± 12.8) for the environment domain, (4.0(3.0–4.0) vs. 3.0(2.0–4.0)) for general health, and (4.0(3.0–4.0) vs. 2.0(2.0–3.0)) for the general QoL, *P* < 0.0001. Patients with TB aged 18–30 years had the highest environmental score compared with the other age groups (*P* = 0.021).

**Conclusions:**

TB had a significant negative impact on QoL, with the physical and psychological domains being the most affected. This finding necessitates strategies to improve QoL of patients with to enhance their compliance to treatment.

**Supplementary Information:**

The online version contains supplementary material available at 10.1186/s12913-023-09381-z.

## Background

Tuberculosis (TB) is a serious infection caused by a bacterium called Mycobacterium tuberculosis, which typically affects the lungs and can also affect other parts of the body, such as the kidney, spine, or brain. TB is considered one of the top 10 causes of death globally, caused approximately 1.6 million deaths in 2021 [1]. Before the coronavirus disease pandemic (COVID-19), TB was the leading cause of death from a single infectious agent, second to human immunodeficiency virus (HIV) [1].

Mycobacterium tuberculosis is thought to infect more than two billion individuals (almost one-fourth of the world’s population) [2]. According to a report by the World Health Organization (WHO) in 2022, there are estimated 10.6 million cases of TB reported worldwide in 2021 compared to 10.1 million (95% UI:9.5–10.7 million) in 2020 [1]. The global average incidence of TB is 189 cases per 100,000 people in 2021. More than (90.0%) of cases occur in low- and middle-income countries (LMIC) [3]. More men than women or children were affected (56.5%, 32.5%, and 11.0%, respectively) [1]. The Eastern Mediterranean Region (EMR) ranks third with 112 cases per 100,000 population per year after Africa and Southeast Asia, which are placed in the first and second positions, respectively. It is estimated that about 11,000 people got TB in Egypt in 2021 with an incidence rate of 10 cases / 100,000 people [1].

TB is an airborne disease transmitted through the air from the infected person while coughing or speaking.[1] Exposure to mycobacteria tuberculosis does not result in TB infection in all exposed population [4]. Most people who are exposed to bacteria can stop bacterial replication and heal themselves or develop latent TB infection (LTBI). Only a few individuals who are exposed to the disease can eventually develop active TB, which can be lethal if not adequately treated [4, 5].

Indeed, TB control services focus on optimizing microbiological cure and using this parameter as an indicator of treatment success. Although this is critical from a public health perspective, it does not effectively explain the physical, mental, and social suffering of patients with TB [5]. TB can cause not only a great impact on the patient’s physical, psychological, and social life but also a remarkable economic challenge [4, 5]. Patients with TB had difficulty in performing their activities of daily living [6]. Previous studies showed that patients with TB experience psychosocial dissatisfaction and social stigma as a result of their disease [7–9]. Furthermore, they reported dissatisfaction with their general health and they showed poor quality of life (QoL) as a result of diminished functioning status, adverse effects of treatment, social stigma, and lack of social support [5]. Financial and income sources, family support, and depression are strongly associated with most QoL domains in TB patients [10, 11]. Furthermore, nonadherence to treatment is negatively associated with all QoL domains. Adverse drug effects can prevent treatment completion, suggesting a need to improve drug adherence [11, 12].

QoL, health related quality of life (HRQoL), and health are sometimes used interchangeably [13]. Bowling et al., [14] defined HRQoL as “an optimum level of mental, physical, role (e.g., work, parent, career, etc.), and social functioning, including relationships, and perceptions of health, fitness, life satisfaction, and well-being’’. HRQoL is a multidimensional concept based on the WHO definition of health that existed in 1948. The WHO stated that health is “a state of complete physical, mental, and social well-being and not mere absence of disease or infirmity” [6]. Later, QoL term was introduced to help in determining the patients’ expression of their preferences and values rather than the clinician’s assessment [15]. Yet, QoL should not be used as a synonym for HRQoL, as they are not the same [16]. QoL is a broad and complex multidimensional concept that is defined by the WHO as an “individual’s perception of their position in life in the context of the culture and value systems in which they live and about their goals, expectations, standards, and concerns” [15].

We hypothesized that consideration of TB patients’ QoL during different stages of treatment and the disease process will enable better insight into the patients’ needs, which will help in designing a tailored TB intervention program. Several studies have been performed worldwide on patients with TB to determine their QoL [10, 11]. However, literature is scarce on the QoL of TB patients in the EMR, particularly in Egypt. Therefore, this survey aimed to assess QoL domains, including the social, psychological, environmental, and physical domains of patients treated for TB in the Alexandria Government, Egypt.

## Methodology

### Study design and sampling technique

This cross-sectional study was conducted at chest clinics and hospitals in the Alexandria Governorate. Alexandria is the second-largest city after Cairo and is the largest city on the Mediterranean coast. The city stretches for approximately 40 km (25 miles) along the northern coast of Egypt. It is the fourth largest city in the Arab world, Africa’s ninth largest city, and the world’s 79th -largest urban area by population. In 2022, Alexandria had a population of approximately 5.3 million people. The cluster sample design was considered to increase the precision and representation. The cluster represents the geographic area that contains the TB health facility (hospital or ambulatory care).

TB patients in Egypt are treated at the hospitals and chest clinics of the Ministry of Health and Population (MoHP). Alexandria has nine governmental health facilities where TB-Laboratory tests are carried out and the drug is supplied. They are consisting of seven chest clinics (Mamoura, Bakos, Gomrok, Amreya, Karmoz, Kom El-Shoafa, and Kabary), and two main chest hospitals (Mamoura, Kom El-Shoafa).

Following the calculation of the sample size and determining the total number of clusters, we recorded a list of diagnostic and treatment units (DTU) for each region (administrative zones). Then we designed a random cluster sampling technique, in which both chest clinics and hospitals were sampled randomly in a stepwise manner. Finally, we randomly selected five units to conduct the study, which are: Mamoura chest hospital, Mamoura chest clinic, Bakos chest clinic, Amerya chest clinic, and Kabary chest clinic). The study participants were recruited consecutively from study settings until reaching the required sample size.

### Study participants and sample size

#### Sample size of TB free population

To calculate the QoL of TB free patients we recruited TB-free population from the same study settings to allow bias-free comparison of QoL scores between TB patients and TB- free population. The size of the matched sample obtained by propensity score matching (PSM) was 131. Using G power software, the calculated power of this study was 99.0% as the mean total QoL mean score of patients with TB patients 45.4 ± 14 (obtained from this current study) and the mean total QoL mean score of the normal population 60.0 ± 14.0 [17].

#### Sample size of patients with TB

The sample size of patients with TB was calculated by assuming that the incidence of TB in Egypt is 12 cases per 100,000 people [18] and the total population of Alexandria is 4 million, the mean QoL score among TB patients was 61.5 ± 12.9 [19] using a margin of error of 2.0% and alpha error of 0.05. Cluster design effect 1.02, non-response rate 5.0%, and alpha error of 0.05. The minimum required sample size was 176 rounded to 180 cases. The sample was calculated using select statistical software [20]. The number of patients recruited from each cluster was proportional to cluster presentation.

### Inclusion and exclusion criteria

Eligible participants included adult patients with TB, aged 18 years and older, at any TB-treatment phase (intensive phase, continuation phase treatment), and attended the sampled facilities during the study period. We excluded patients with associated pulmonary diseases such as lung cancer, chronic obstructive pulmonary disease, and asthma. Patients with mental disabilities, deaf, and mute were excluded as well.

### Data collection methods and sources

Structured interviewing questionnaire was used to collect data from participants from November 20, 2021, till the end of June 2022 through face-to-face interview. The interview was conducted at the health facility and took 5–10 min. The research team conducted the interview in a separate room / space to ensure patient privacy. The interviewers have already received an educational session on infection control measures. They conducted the interview in a well-ventilated room and wore an N-95 respirator mask. The questionnaire consisted of four sections; the first section was completed from the patient profile to ensure if he/she was eligible or not. Data collectors gathered the following data from patients’ profile: date of symptom onset, date of diagnosis, treatment phase (intensive or continuation phase), current phase start date, (multidrug resistant (MDR) vs. drug sensitive (DS)). The second section was personal and demographic data including (age, sex, marital status, residence, education level, occupation, history of chronic diseases, and how the TB affected his/her life). Patients were categorized into low (scored < 40.0%), middle (scored 40.0% to < 70.0%), and high income (scored ≥ 70.0%). The third section included socioeconomic level related questions including (mother education, father education, mother work, fathers work, computer use, per capita income, family size, crowding index, sewage disposal, refuse disposal).[21] The fourth section is the Arabic-validated WHOQOL-BREF instrument [22]. The WHOQOL-BREF consists of 26 items, two items for evaluating general QoL and general health, and 24 items for assessing QoL in four domains, namely physical domain (seven items), psychological domain (six items), social relationship domain (three items), and environmental domain (eight items). The tool follows a scoring system, where each question is rated on a 5-point Likert scale, ranging from 1 (very poor/very dissatisfied/none/ never) to 5 (very good/very satisfied/extremely/always). Then the scores of all four domains were summed and scaled positively, transformed to a 0–100 scale with higher scores indicating better QoL [23]. The estimated acceptable values of the QoL domains in the general population are as follows: physical health QoL = 73.5 ± 18.1, psychological QoL = 70.6 ± 14.0, social relationship QoL = 71.5 ± 18.2, and environmental QoL = 75.1 ± 13.0 [17]. Respondents whose scores were above these thresholds were classified as having good quality, while those with scores below the thresholds were classified as having poor quality. We followed the Checklist for Strengthening the Reporting of Observational studies in Epidemiology (STROBE S1) [24].

### Selection of the control

Several combinations of confounders which were thought to be of great impact were used for matching and the best results were obtained by using these 4 confounders: age groups, gender, presence of chronic disease other than TB, and residence. Supplementary Table S2 shows the results of the balanced covariate distribution resumed after PSM; all covariates showed an improved balance between the matched groups confirmed by insignificant X^2^ test statistics, except for the age groups that turned significant after matching. Though matched groups were considered balanced as this was the best balance available by the sample obtained, and these matched groups were used for purposes of comparisons only between TB patients and TB-free population to allow for the clear highlighting of the difference caused by TB disease and were not used for associations analysis. The balance hypothesis was supported by the graphs S3, 4 obtained by comparing the predicted probability distributions between matched and unmatched groups.

### Data analysis

Descriptive analysis was used to report the demographic characteristics of the participants. Categorical variables were presented as percentages and continuous variables as means ± standard deviation [25]. We used median and interquartile range (IQR) to describe non-parametric quantiative data. Assumptions of normal distribution were explored with the Kolmogorov-Smirnov test and visual inspections of the histograms. We used the independent t-test and Mann–Whitney U test Mann to investigate the differences between QoL domains. The level of significance was set at *P* < 0.05. Statistical analysis was performed using SPSS software version 25 software.

## Results

### The QoL of patients with TB compared with TB free population (n = 262)

The total QoL score of the TB free population was significantly higher than that of TB patients (60.6 ± 14.3 vs 44.8 ± 13.6), *P* < 0.0001). The scores of the four domain of the TB free population group were significantly higher than that of TB patients’ group. (65.0 ± 17.5 vs 42.4 ± 17.8) for the physical domain, (59.2 ± 13.6 vs 41.9 ± 15.1) for the psychological domain, (61.8 ± 19.9 vs 50.3 ± 20.6) for the social domain, and (56.3 ± 19.3vs 44.5 ± 12.8) for the environment domain respectively. *P* < 0.0001. Similarly, the median(IQR) scores of general health and the general QoL, were significantly higher among the TB free patients compared with patients with TB (4.0(3.0–4.0) vs 3.0(2.0–4.0), and 4.0(3.0–4.0) vs 2.0(2.0–3.0) respectively) (*P* < 0.0001) (Table 1).

**Table 1:** Comparison of the QoL scores between TB patients and normal population matched groups

	TB patients N = 131 mean ± SD	TB free population N = 131 mean ± SD	Test statistics, P value
Physical domain	42.4 ± 17.8	65.0 ± 17.5	10.4, 0.01^*t^
Psychological domain	41.9 ± 15.1	59.2 ± 13.6	9.7, 0.001^*t^
Social domain	50.3 ± 20.6	61.8 ± 19.9	4.6, 0.001^*t^
Environmental domain	44.5 ± 12.8	56.3 ± 19.3	5.8, 0.001^*t^
Total QoL score	44.8 ± 13.6	60.6 ± 14.3	9.1, 0.001^*t^
General health median (IQR)	3.0(2.0–4.0)	4(3.0–4.0)	4740.5, 0.001^*u^
General QoL median (IQR)	2.0(2.0–3.0)	4.0(3.0–4.0)	3272.5, 0.001^*u^

^(t)^Independent t-test, ^(u)^Mann–Whitney U test, * significant p-value

### Patients with TB characteristics (n = 180)

A total of 180 patients participated in the study, among them, 74.4% were males, 54.4% were married, 60.0% were 18.0–40.0 years with a median value of 37.0 (29.0–48.0), 83.3% lived in urban areas, 31.7% were illiterate, 69.5% reported that income was not enough, 58.9% had a crowding index of > 2, and 71.7% were of medium socioeconomic class (Table 2).

**Table 2:** Sociodemographic and health-related characteristics of TB patients

Variables	(n = 180)
Sex	
Male Female	134(74.4) 46(25.6)
Age in years	
18–30 31–40 41–50 51–60 61–75 Median (IQR)	52(28.9) 56(31.1) 37(20.6) 24(13.3) 11(6.1) 3.07 (29.0–48.0)
Marital status	
Married Single Divorced Widow	98(54.4) 68(37.8) 9(5.0) 5(2.8)
Residence	
Urban Rural	150(83.3) 30(16.7)
Education level	
Do not read/write (illiterates) Read and write Junior Middle Senior Graduate	35(19.4) 13(7.2) 28(15.6) 40(22.2) 45(25.0) 19(10.6)
Income per capita	
Not enough and loan not repaid Not enough and big loan Not enough and small loan Enough Enough and saving	37(20.6) 33(18.3) 55(30.6) 54(30) 1(0.6)
Family size	
1–4 ≥ 5	109(60.6) 71(39.4)
Crowding index	
< 2 2–4 ≥ 4	106(58.9) 62(34.4) 12(6.7)
Socioeconomic class	
Low < 40% Medium 40 to < 70% High ≥ 70%	41(22.8) 129(71.7) 10(5.6)

Regarding health status, 71.7% of the sample suffered from pulmonary TB, 55.0% were in the continuation phase of treatment, 52.2% suffered from TB for ≤ 6 months (start of symptoms till the time of interview), 10.0% were treated from MDR-TB, 36.7% were suffering from other comorbidities, 68.3% of them reported food insecurity, 69.4% lost their jobs, 15.6 felt stigmatized and exposed to social ostracism, while 5.6% lost their partners (Table 3).

**Table 3:** TB status and characteristics of patients

Variable	(n = 180)
TB type	
Pulmonary TB Extrapulmonary TB	129(71.7) 51(28.3)
Treatment phase	
Intensive phase Continuation phase	81 (45.0) 99(55.0)
Disease duration	
≤ 6 months > 6 months	94(52.2) 86(47.8)
MDR	
MDR Not MDR	18(10.0) 162(90.0)
Chronic disease	
No Yes	114(63.3) 66(36.7)
Impact of TB	
No impact Loss of food Loss of work Social ostracism Lost their partners	27 (15.0) 123(68.3) 125(69.4) 28(15.6) 10(5.6)

### Measured QoL score among the patients with TB (n = 180)

**Fig. 1:**
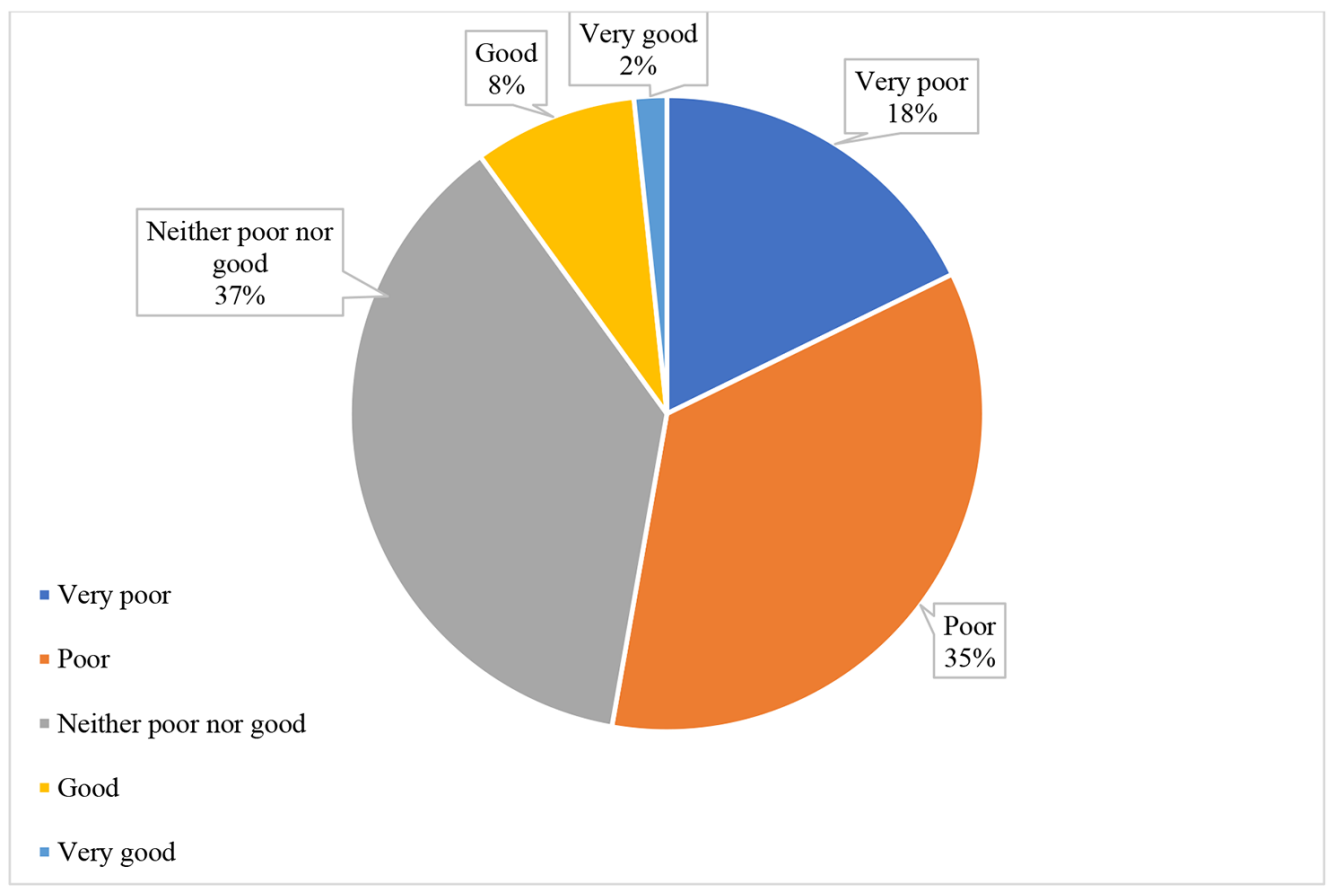
Patients’ perception of their general quality of life

**Fig. 2:**
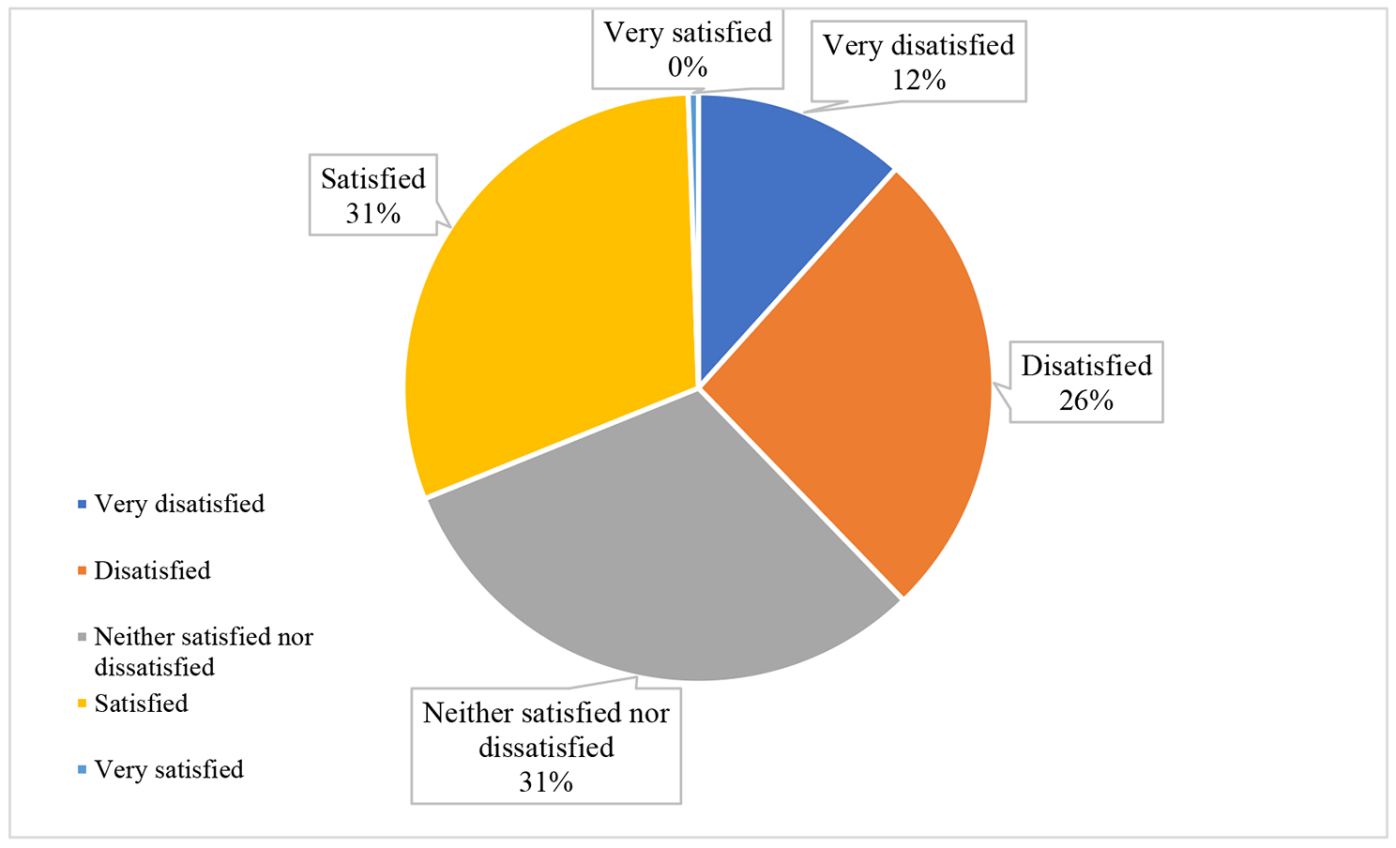
Patient perceptions of their general health

**Fig. 3:**
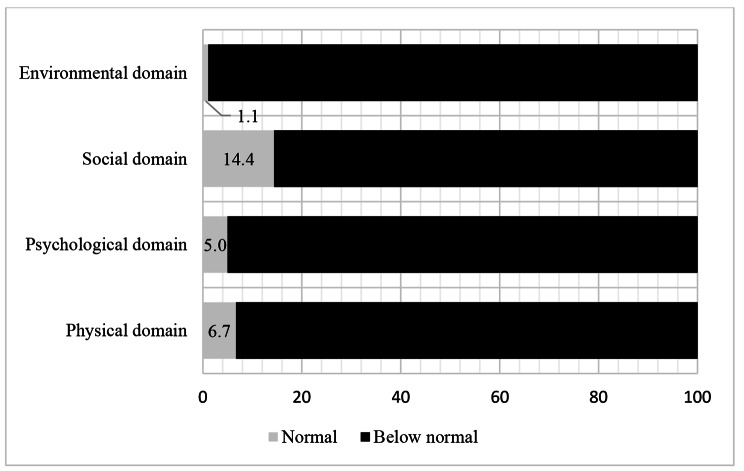
Proportion of normal QoL scores among TB patients

The mean score of all QoL domains of the total studied samples was 45.4 ± 14 with 42.8 ± 16.1 for the psychological domain, 42.8 ± 18.8 for the physical domain, 50.1 ± 20.5 for the social domain, and 45.4 ± 13.1 for the environmental domain. The median score for the general QoL was 2.0 (2.0–3.0) (Fig. 1), while the median score for the general health domain was 3.0 (2.0–4.0) (Fig. 2). About 14.0% of patients had normal social domain, 6.7% had normal physical domain, 5.0% had normal psychological domain, and 1.1 had normal environmental domain. (Fig. 3)

Male participants had lower mean scores of physical and psychological domains of QoL than females (42.4 ± 18.6 vs. 43 ± 19.7) vs. (42.6 ± 16 vs. 43.4 ± 16.8), however, these differences were not statistically significant. The age group between 18 and 30 years had the highest scores in the physical, psychological and environmental domains, the significant difference was only observed for the environmental domain. Compared to married participants, singles had significantly higher QoL scores in all domains. Patients who lived in urban areas had a higher score in the physical domain than those who lived in rural areas (43.6 ± 18.8 vs. 38.5 ± 18.3), while they had lower scores in the social and environmental domains (49.9 ± 21.2 vs53.6 ± 16.2) and (49.9 ± 21.2 vs. 53.6 ± 16.2) respectively. None of these differences were statistically significant. The scores of psychological domains were comparable in both areas (42.9 ± 16.5 vs. 42.4 ± 14.4). Regarding the university graduates’ patients were of the highest scores in the physical and psychological domains (51.5 ± 20.9 and 45.8 ± 15.4 respectively), while ignorant patients had the lowest scores (38.4 ± 18.1), patients who can only read and write had surprisingly the highest scores in the social domains (57.1 ± 12.7). Scores of the environmental domain were comparable with top score to patients who stopped their education at the junior level. Patients of medium socioeconomic class had the greatest scores in all domains). However, these differences were not significant. Regarding health status of the patients, participants of extrapulmonary TB and who in the continuation phase of treatment had better scores of all domains of QoL than pulmonary TB and patients during the intensive phase, yet this difference was not significant. All QoL domain scores were comparable (almost equal) in patients with disease durations shorter than and longer than 6 months. Patients of MDR TB had higher scores in physical and psychological domains (44.0 ± 20.4 vs. 42.6 ± 18.7) and (43.1 ± 16.8 vs. 42.6 ± 18.7) respectively, while they had the lowest scores in the social and environmental scores comparing to non-MDR-TB (48.6 ± 20.7 vs. 50.7 ± 20.5) and (47.3 ± 13.5 vs. 44.4 ± 12.8), but this was not considered statistically significant. Patients with chronic diseases other than TB had higher scores in all QoL domains than those who suffered from TB especially in environmental and social domains, yet this difference were not statistically significant (Table 4).

**Table 4:** Sociodemographic and scores means of QoL domains of TB patients

	Physical domain Score	Psychological Domain score	Social Domain score	Environmental Domain score
Gender				
Male (134) Female (46) P value	42.4 ± 18.6 43 ± 19.7 0.67	42.6 ± 16 43.4 ± 16.8 0.79	20.4 ± 1.8 20.8 ± 3.1 0.70	45.5 ± 12.6 45.3 ± 14.6 0.90
Age groups:				
18–30 (52) 31–40(56) 41–50(37) 51–60(24) 61–75 (11) P value	45.9 ± 19.9 40.1 ± 15.6 41.5 ± 19.8 44.2 ± 20.2 42.5 ± 22.5 0.58	46.3 ± 18.5 39.5 ± 13 43 ± 17.2 42.5 ± 14.5 42.8 ± 17.7 0.29	55.1 ± 20.7 47.2 ± 18.8 50.2 ± 22.4 46.5 ± 19.3 55.3 ± 21.2 0.23	50.2 ± 14.9 41.9 ± 10.9 45 ± 13.9 44.5 ± 10.7 43.4 ± 11.1 0.02*
Marital status				
Married (98) Single (82) P value	42.2 ± 18.9 43.5 ± 18.7 0.65	41.4 ± 16.0 44.6 ± 16.2 0.19	49.8 ± 20.9 51.3 ± 20.0 0.63	44.0 ± 12.2 47.1 ± 14.0 0.11
Residence				
Urban (150) Rural (30) P value	43.6 ± 18.8 38.5 ± 18.3 0.17	42.9 ± 16.5 42.4 ± 14.4 0.86	49.9 ± 21.2 53.6 ± 16.2 0.37	49.9 ± 21.2 53.6 ± 16.2 0.31
Education level				
Senior (45) Middle (40) Ignorant (35) Junior (28) Graduate (19) Read and write (13) P value	43.7 ± 19.6 41.6 ± 19.7 38.4 ± 18.1 41.2 ± 14.3 51.5 ± 20.9 45.9 ± 19.1 0.24	44.2 ± 17.3 45.1 ± 16.7 39.0 ± 16.4 39.3 ± 15.7 45.8 ± 15.4 44.6 ± 9.5 0.40	49.6 ± 19.9 48.5 ± 20.6 48.6 ± 24.4 53.0 ± 17.5 52.2 ± 23.2 57.1 ± 12.7 0.76	45.5 ± 14,8 46.5 ± 12.3 43.3 ± 12.7 47.4 ± 13.4 44.1 ± 13.6 45.4 ± 9.9 0.85
Socioeconomic class				
Low (41) Medium (129) High (10) P value	39.8 ± 18.4 43.7 ± 17.9 42.5 ± 30.0 0.51	40.9 ± 43.7 43.7 ± 15.9 39.6 ± 22.2 0.50	50.4 ± 20.2 51.1 ± 20.2 43.3 ± 25.1 0.51	45.0 ± 12.1 45.9 ± 13.4 41.3 ± 13.6 0.55
TB type				
Pulmonary TB (129) Extrapulmonary TB (51) P value	42.2 ± 19.1 44.3 ± 18.0 0.49	42.5 ± 16.3 43.5 ± 15.8 0.71	49.7 ± 20.8 52.5 ± 19.8 0.43	45.1 ± 13.3 46.2 ± 12.8 0.60
Disease duration				
≤ 6 months (94) > 6 months (86) P value	42.7 ± 19.9 42.8 ± 17.7 0.98	43.3 ± 15.7 42.3 ± 16.6 0.68	50.6 ± 22.7 50.4 ± 17.8 0.93	45.9 ± 14 44.9 ± 12.1 0.61
Treatment phase				
Intensive phase(81) Continuation phase (99) P value	40.9 ± 20.5 44.3 ± 17.2 0.22	41.1 ± 16.7 44.2 ± 15.6 0.20	49.3 ± 22.3 51.5 ± 18.9 0.47	43.9 ± 14,1 46.7 ± 12.1 0.17
MDR				
MDR (18) Not MDR (162) P value	44.0 ± 20.4 42.6 ± 18.7 0.67	43.1 ± 16.8 42.6 ± 18.7 0.95	48.6 ± 20.7 50.7 ± 20.5 0.68	44.4 ± 12.2 45.5 ± 13.2 0.74
Chronic diseases				
Present (64) Absent (116) P value	43.5 ± 20.8 42.4 ± 17.7 0.69	44.3 ± 17.1 42.0 ± 15.6 0.37	53.3 ± 20.6 49.0 ± 20.3 0.18	47.3 ± 13.5 44.4 ± 12.8 0.15

## Discussion

This multicenter cross-sectional study using the WHOQOL-BREF instrument revealed that TB had a significant influence on several QoL dimensions. This study found that in addition to general health and general QoL scores, the four domains of QoL of the patients with TB were significantly lower than those of the non-TB population QoL. However, the scores of normal population were lower than those reported in previous studies. This mild decrease in normal healthy population QoL may be due the deletrious effect of COVID-19 [7, 26, 27], especially the difference in physical activities, it could be due to the change in lifestyle with implementing different public health and social measures to contain the COVID-19 pandemic [28]. Copying with these findings, Taiwanese patients with TB had a greater impairment in the physical, environmental, and psychological domains QoL than healthy individuals [8]. The majority of participants with TB in the current study thought their health was fair. These results are consistent with the finding of a research carried out in Indonesia, where just 14.0% of TB patients reported being satisfied with their QoL [7]. We found that the domain of general perceptions of QoL of TB patients had the lowest scores, followed by the physical and psychological domains. The highest QoL score had been seen for the social domain (50.1 ± 20.5). Similarly, Indonesian patients with TB perceived dissatisfaction with all domains of their QoL except for the psychological domain. The authors claimed that religious background of the patients might have an impact on this finding [7]. In the same vein, in Eritrea, the environmental and physical domains of QoL were the most affected among patients with TB regardless of the type of therapy received [rifampicin-multidrug-resistant vs. drug-susceptible [29], while the psychological domain was the least affected [30]. Patients with TB experienced social discrimination and internalized stigma related to having TB [8]. Similarly, the social domain of QoL was affected more than the other domains in Ethiopian patients with TB; however, non-adherence to treatment was negatively associated with all HRQoL domains [31]. Indeed, in general, TB decreases QoL due to different factors such as; low activity level in carrying out everyday tasks, insufficient rest and sleep, increased reliance on medications, increased pain and discomfort, insufficient energy and mobility, and poor capacity for work are all implied [32]. Several symptoms, including cough, fever, weight loss, and exhaustion, were the root causes of reduced physical scores. Negative feelings, nightmares, suicidal attacks, and depression as the side effects of TB or its medications can affect the psychological domain of QoL. The sense of security, available funds, access to medical treatment, leisure activities, the environment at home, and transportation are all related to the domain of environmental health. TB patients may become isolated from other community members as a result of the social stigma associated with the condition [33]. It is worth noting that patients treated for TB reported improvement in the mean score of vitality and mental health from baseline to the two-month visit, and improvement in the physical role from baseline to the four-month visit [34]. In Iran, TB patients had lower HRQoL scores than controls before starting treatment. The patients’ scores increased significantly after two months of treatment, however the difference between two and six months following treatment was not significant [9]. This highlights the need for early treating patients with TB to improve their QoL. This may indirectly improve patient compliance to the prescribed treatment.

In particular, many modifiable and nonmodifiable factors have been found to be related to the perceived QoL and overall perceived health status, which vary relatively between countries. We found that age has a significant impact on the environmental domain of QoL and couldn’t be matched in the propensity score model. In concordance with our finding, Juliasih et al., [32], reported that the age category of 18–30 years has the highest QoL score and surprisingly the least QoL scores was observed among patients aged 31–40 years. In terms of sociodemographic factors, patients with TB are more likely to be males, less educated, and have a low economic status [7, 8, 31, 35]. However, in the current study, factors like sex, marital status, education level, residence and socioeconomic level had no significant effect on the QoL of patients with TB.

Based on the result of meta-analysis of more than 12 thousand patients with TB, about 43.0% may incur catastrophic costs that exceed 20.0% of their income [36]. Higher proportion (59.1%) of the Egyptians incurred these costs [37, 38]. We did not find significant association between patient income level and QoL. On the other hand, income loss was strongly associated with worse QoL domains score in many studies [7, 8, 30, 35]. As more than 90.0% of the studied population were of low-and middle-income, so we could not assess the association between high- and low-income patients with QoL. This objective can be more precisely described by other studies that stratify patients based on their income level.

In the current study, 15.6% of the studied patients reported stigma due to their diseases. In fact, the social stigma associated with TB can also affect patients’ psychological well-being [35]. Previous studies recommended a number of interventions to reduce the stigma associated with TB, including the TB club program in Africa run by healthcare professionals, which offers social support and compliance/side effect monitoring to TB patients. Furthermore, health authorities should provide education programs targeting community members of the at-risk community to change their false perceptions about the disease. Finally, patient counseling can effectively reduce the negative impact of this stigma. [39–42].

### Strengths and limitations

To the best of our knowledge, this is the first study that addressed the QoL among patients with TB in Egypt. In addition, we included all treatment setting (outpatient and inpatient clinics) and patients at different treatment phases (intensive or continuation phases) with different diagnoses (pulmonary and extrapulmonary TB) and treatment regimens (drug sensitive and drug resistant) through face-to-face interview. We used probability sampling method (cluster design) to ensure the generalization of the findings. However, the study had many limitations including the inherent limitations of the adopted study design (cross-sectional survey) such recall bias, inability to assess causality, and incapability to study the outcome across different time periods. Second, we did not assess QoL at different time points to describe the change across time concurrently with treatment. Third, we included only adults, future studies are required to include children and youth to investigate the effect of TB on their QoL. Fourth, patients with TB were not matched with TB free population regarding age. Finally, due to the ongoing pandemic and its related public health measures we conducted our study in one government, so larger national studies may be crucial to provide more representation of patients with TB in Egypt.

## Conclusions

In conclusion, most TB patients have unsatisfactory QoL scores in different domains (physical, psychological, social and environmental) compared to TB free population. This may highlight the importance of monitoring QoL as a part of the evaluation of response to long term treatment and medical care for patients with TB to ensure case improvement and better treatment compliance.

## Electronic supplementary material

Below is the link to the electronic supplementary material.

Supplementary Material 1

Supplementary Material 2

## Data Availability

The data sets used and/or analyzed during the current study are available by emailing the corresponding author on reasonable request.

## List of Abbreviations

AIDS: Acquired immunodeficiency syndrome
COVID-19: Corona virus disease 2019
DS: Drug-susceptible
DTU: Diagnostic and treatment units
EMR: Eastern Mediterranean Region
HIV: Human immunodeficiency virus
HRQoL: Health-Related Quality of Life
IQR: Inter-quartile range
LTBI: Latent TB infection
MDR: Multi-drug resistant
MoHP: Ministry of Health and Population
MoHP-REC: Ministry of Health and Population Research Ethics Committee
PSM: Propensity score matching
SD: Standard deviation
TB: Tuberculosis
WHO: World Health Organization
