# Supplementary material for: Survey to measure the quality of life of patients with tuberculosis in Alexandria, Egypt: a cross-sectional study
Source: BMC Health Serv Res. 2023 May 24;23:534. doi: 10.1186/s12913-023-09381-z (PMC10208181; doi:10.1186/s12913-023-09381-z)
Supplement: Supplementary file 1 — Supplementary Material 1 [file 12913_2023_9381_MOESM1_ESM.docx]

**Survey To Measure The Quality of Life of Patients With Tuberculosis In Alexandria, Egypt: A Cross-Sectional Study**

Esraa Abdellatif Hammouda* (1), Wahib Fayez Gobran (2), Reem Mohamed Tawfeek (3), Ola Fahmy Esmail (4), Rasha Ashmawy (5), Naglaa Youssef (6), Ramy Mohamed Ghazy (7)

**Review and approval of the study protocol were obtained by the Ethical committee of the High Institute of Public Health, Alexandria University (Com.No/Oct. 26/10/ 2021)**

1. **Interview date: / /**
2. **Health facility name:**

| **1.Maamora Chest Hospital** | **2. Maamora Chest Center** |
| --- | --- |
| **3.Bakoos Chest Center** | **4.Alamrya Chest Center** |
| **5.Elkabary Chest Center** |  |

**Section I: Medical history**

1. **Onset date of symptoms: / /**
2. **First diagnostic test date: / /**
3. **TB type:**

| **1. Pulmonary TB** | **2. Extra-pulmonary TB** |
| --- | --- |

1. **The current treatment phase:**

| **1. Intensive phase** | **2.** c**continuation phase** |
| --- | --- |

1. **Current phase start date: / /**
2. **Are you on a multidrug-resistant treatment regimen?**

| **1.Yes** | **2. No** |
| --- | --- |

**Section II: Personal and demographic data**

1. **Gender: 1. Male 2. Female**
2. **Age:**
3. **Marital Status:**

| 1. **Married** | 1. **Single** |
| --- | --- |
| 1. **Widowed** | 1. **Divorced** |

1. **Residence**

| 1. **Rural** | 1. **Urban** |
| --- | --- |

1. **Patient education:**

| 1. **Illiterate/read and write** | 1. **Literate certificate** |
| --- | --- |
| 1. **Primary** | 1. **Preparatory** |
| 1. **Secondary** | 1. **University** |
| 1. **Postgraduate** |  |

1. **Do you suffer from other chronic diseases?**

|  | \| 1. **Yes** \| 1. **No** \| \| --- \| --- \| |
| --- | --- | --- | --- |

1. **If the answer to the previous question is yes, specify those diseases.**

| 1. **Hypertension** | 1. **Diabetes Mellitus** |
| --- | --- |
| 1. **Cardiovascular disease** | 1. **Chest diseases** |
| 1. **Renal or hepatic diseases** | 1. **Rheumatic or autoimmune diseases** |
| 1. **psycho-neurological diseases** | 1. **Disabilities** |
| 1. **Deafness or blindness** | 1. **Others** |

1. **TB effect on your social life**

| 1. **Food deficiency** | 1. **Divorce or partner separation** |
| --- | --- |
| 1. **Social Stigma** | 1. **Loss of work or education** |
| 1. **Was not affected** |  |

**Section III: Socioeconomic status evaluation**

1. **Mother’s education**

| **1. Illiterate/read and write** | **2. Literate certificate** |
| --- | --- |
| **3. Primary** | **4. Preparatory** |
| **5. Secondary** | **6. University** |
| **7. Postgraduate** |  |

1. **Father’s education**

| **1. Illiterate/read and write** | **2. Literate certificate** |
| --- | --- |
| **3. Primary** | **4. Preparatory** |
| **5. Secondary** | **6. University** |
| **7. Postgraduate** |  |

1. **Mother’s work**

| **Yes** | 1. **No** |
| --- | --- |

1. **Father’s work**

| 1. **Yes** | 1. **No** |
| --- | --- |

1. **Computer use**

| **1.Never** | **2. Sometimes** |
| --- | --- |
| **3. Lot of times** |  |

1. **Per-capita income**

| **2. Not enough+big loan** | **1. Not enough+loan not repaid** |
| --- | --- |
| **4. Enough only** | **3. Not enough+small loan** |
|  | **5. Enough and saving** |

1. **Family size**

| **1. ≥7** | **2. 6** |
| --- | --- |
| **3.5** | **4. ˂ 5** |

1. **Crowding index**

| **1. ≥4** | **2. 2–** |
| --- | --- |
| **3. <2** |  |

1. **Sewage disposal**

| **1.Yes** | **2. No** |
| --- | --- |

1. **Refuse disposal**

| **1.Yes** | **2. No** |
| --- | --- |

**Section IV: The WHOQOL-BREF**

1. **How would you rate your quality of life?**

| **1. Very poor** | **2. Poor** |
| --- | --- |
| **3. Neither poor nor good** | **4. Good** |
| **5. Very good** |  |

1. **How satisfied are you with your health**

| **1. Very dissatisfied** | **2. Fairly Dissatisfied** |
| --- | --- |
| **3. Neither satisfied nor dissatisfied** | **4. Satisfied** |
| **5. Very satisfied** |  |

**The following questions ask about how much you have experienced certain things in the last two weeks.**

1. **To what extent do you feel that physical pain prevents you from doing what you need to do?**

| **1. Not at all** | **2. A Small amount** |
| --- | --- |
| **3. A Moderate amount** | **4. A great deal** |
| **5. An Extreme amount** |  |

1. **How much do you need any medical treatment to function in your daily life?**

| **1. Not at all** | **2. A Small amount** |
| --- | --- |
| **3. A Moderate amount** | **4. A great deal** |
| **5. An Extreme amount** |  |

1. **How much do you enjoy life?**

| **1. Not at all** | **2. A Small amount** |
| --- | --- |
| **3. A Moderate amount** | **4. A great deal** |
| **5. An Extreme amount** |  |

1. **To what extent do you feel your life to be meaningful?**

| **1. Not at all** | **2. A Small amount** |
| --- | --- |
| **3. A Moderate amount** | **4. A great deal** |
| **5. An Extreme amount** |  |

1. **How well are you able to concentrate?**

| **1. Not at all** | **2. Slightly** |
| --- | --- |
| **3. Moderately** | **4. Very** |
| **5. Extremely** |  |

1. **How safe do you feel in your daily life?**

| **1. Not at all** | **2. Slightly** |
| --- | --- |
| **3. Moderately** | **4. Very** |
| **5. Extremely** |  |

1. **How healthy is your physical environment?**

| **1. Not at all** | **2. Slightly** |
| --- | --- |
| **3. Moderately** | **4. Very** |
| **5. Extremely** |  |

1. **Do you have enough energy for everyday life?**

| **1. Not at all** | **2. Slightly** |
| --- | --- |
| **3. Somewhat** | **4. To a great extent** |
| **5. Completely** |  |

1. **Are you able to accept your bodily appearance?**

| **1. Not at all** | **2. Slightly** |
| --- | --- |
| **3. Somewhat** | **4. To a great extent** |
| **5. Completely** |  |

1. **Have you enough money to meet your needs?**

| **1. Not at all** | **2. Slightly** |
| --- | --- |
| **3. Somewhat** | **4. To a great extent** |
| **5. Completely** |  |

1. **How available to you is the information you need in your daily life?**

| **1. Not at all** | **2. Slightly** |
| --- | --- |
| **3. Somewhat** | **4. To a great extent** |
| **5. Completely** |  |

1. **To what extent do you have the opportunity for leisure activities?**

| **1. Not at all** | **2. Slightly** |
| --- | --- |
| **3. Somewhat** | **4. To a great extent** |
| **5. Completely** |  |

1. **How well are you able to get around physically?**

| **1. Not at all** | **2. Slightly** |
| --- | --- |
| **3. Moderately** | **4. Very** |
| **5. Extremely** |  |

**The following questions ask you to say how good or satisfied you have felt about various aspects of your life over the over the last two weeks.**

1. **How satisfied are you with your sleep?**

| **1. Very dissatisfied** | **2. Fairly Dissatisfied** |
| --- | --- |
| **3. Neither satisfied nor dissatisfied** | **4. Satisfied** |
| **5. Very satisfied** |  |

1. **How satisfied are you with your ability to perform your daily living activities?**

| **1. Very dissatisfied** | **2. Fairly Dissatisfied** |
| --- | --- |
| **3. Neither satisfied nor dissatisfied** | **4. Satisfied** |
| **5. Very satisfied** |  |

1. **How satisfied are you with your capacity for work?**

| **1. Very dissatisfied** | **2. Fairly Dissatisfied** |
| --- | --- |
| **3. Neither satisfied nor dissatisfied** | **4. Satisfied** |
| **5. Very satisfied** |  |

1. **How satisfied are you with yourself?**

| **1. Very dissatisfied** | **2. Fairly Dissatisfied** |
| --- | --- |
| **3. Neither satisfied nor dissatisfied** | **4. Satisfied** |
| **5. Very satisfied** |  |

1. **How satisfied are you with your personal relationships?**

| **1. Very dissatisfied** | **2. Fairly Dissatisfied** |
| --- | --- |
| **3. Neither satisfied nor dissatisfied** | **4. Satisfied** |
| **5. Very satisfied** |  |

1. **How satisfied are you with your sex life?**

| **1. Very dissatisfied** | **2. Fairly Dissatisfied** |
| --- | --- |
| **3. Neither satisfied nor dissatisfied** | **4. Satisfied** |
| **5. Very satisfied** |  |

1. **How satisfied are you with the support you get from your friends?**

| **1. Very dissatisfied** | **2. Fairly Dissatisfied** |
| --- | --- |
| **3. Neither satisfied nor dissatisfied** | **4. Satisfied** |
| **5. Very satisfied** |  |

1. **How satisfied are you with the conditions of your living place?**

| **1. Very dissatisfied** | **2. Fairly Dissatisfied** |
| --- | --- |
| **3. Neither satisfied nor dissatisfied** | **4. Satisfied** |
| **5. Very satisfied** |  |

1. **How satisfied are you with your access to health services?**

| **1. Very dissatisfied** | **2. Fairly Dissatisfied** |
| --- | --- |
| **3. Neither satisfied nor dissatisfied** | **4. Satisfied** |
| **5. Very satisfied** |  |

1. **How satisfied are you with your transport?**

| **1. Very dissatisfied** | **2. Fairly Dissatisfied** |
| --- | --- |
| **3. Neither satisfied nor dissatisfied** | **4. Satisfied** |
| **5. Very satisfied** |  |

**The following question refers to how often you have felt or experienced certain things in the last two weeks.**

1. **How often do you have negative feelings such as blue mood, despair, anxiety or depression?**

| **1. Never** | **2. Infrequently** |
| --- | --- |
| **3. Sometimes** | **4. Frequently** |
| **5. Always** |  |
