# Supplementary material for: Survey to measure the quality of life of patients with tuberculosis in Alexandria, Egypt: a cross-sectional study
Source: BMC Health Serv Res. 2023 May 24;23:534. doi: 10.1186/s12913-023-09381-z (PMC10208181; doi:10.1186/s12913-023-09381-z)
Supplement: Supplementary file 2 — Supplementary Material 2 [file 12913_2023_9381_MOESM2_ESM.doc]

**Supplementary materials for the manuscript:**

Title: **Survey To Measure The Quality of Life of Patients With Tuberculosis In Alexandria, Egypt: A Cross-Sectional Study**

**Esraa Abdellatif Hammouda* ^(1)^, Wahib Fayez Gobran ^(2)^, Reem Mohamed Tawfeek ^(3)^, Ola Fahmy Esmail ^(4)^, Rasha Ashmawy ^(5)^, Naglaa Youssef ^(6)^, Ramy Mohamed Ghazy^(7)^**

1. Department of Clinical Research, El-Raml pediatric hospital, Ministry of Health and Population, Alexandria, Egypt.

ORCID: 0000-0003-3269-6623 – email: [Hiph.eabdellatif@alexu.edu.eg](mailto:Hiph.eabdellatif@alexu.edu.eg)

1. Director of Chest Diseases, Ministry of Health and Population, Alexandria, Egypt.

email: [Wahib_gs@yahoo.com](mailto:Wahib_gs@yahoo.com)

1. Ministry of Health and Population, Alexandria, Egypt.

ORCID: [0000-0002-8493-8850](https://orcid.org/0000-0002-8493-8850), email: [Reemtawfeek@hotmail.com](mailto:Reemtawfeek@hotmail.com)

1. Egyptian Drug Authority, email: [Olafahmy@alexu.edu.eg](mailto:Olafahmy@alexu.edu.eg)
2. Department of Clinical Research, Maamoura Chest Hospital, Ministry of Health and Population, Alexandria, Egypt.

ORCID: 0000-0003-2689-5968, email: [mri.rasha.m.informatics19@alexu.edu.eg](mailto:mri.rasha.m.informatics19@alexu.edu.eg)

1. Medical-Surgical Nursing Department, Faculty of Nursing, Cairo University.

ORCID: [0000-0002-0368-1759](https://orcid.org/0000-0002-0368-1759) – email: [youssef_naglaa@cu.edu.eg](mailto:youssef_naglaa@cu.edu.eg)

1. Tropical Health Department, High Institute of Public Health, Alexandria University, Egypt,

ORCID: 0000-0001-7611-706X – email: [ramy_ghazy@alexu.edu.eg](mailto:ramy_ghazy@alexu.edu.eg)

**^*^Correspondence:**

Esraa Abdellatif Hammouda

Clinical research department, El-Raml Pediatric Hospital. 6 Mohamed Farid Winget St. Bolkely, Alexandria, Egypt.

Phone: + (2) 01224054671

Email: [hiph.eabdellatif@alexu.edu.eg](mailto:hiph.eabdellatif@alexu.edu.eg)

**Supplementary 1 (S1): STROBE Statement - checklist for our study**

| Strobe recommendation | Item No | Our study | Page No |
| --- | --- | --- | --- |
| Title and abstract | | | |
| (*a*) Indicate the study’s design with a commonly used term in the title or the abstract | 1 | (a)  Title: Survey to measure the quality of life of patients with tuberculosis in Alexandria, Egypt: a cross-sectional study  Abstract: Study design included in abstract:  “Methods: This cross-sectional study was conducted in chest clinics and main chest hospitals in Alexandria, Egypt. A structured interview questionnaire was used to collect data from participants through face-to-face interviews from November 20, 2021, until the June 30, 2022. We included all adult patients aged 18 years or above during the intensive or continuation phase of treatment. The World Health Organization (WHO) WHOQOL-BREF instrument was used to measure QoL, which includes the physical, psychological, social relationships, and environmental health domains. Using propensity score matching, a group of TB free population was recruited from the same setting and completed the questionnaire.” | Page 1 |
| (*b*) Provide in the abstract an informative and balanced summary of what was done and what was found |  | (b) Structured abstract provided. | Page 1-2 |
| Introduction | | | |
| Background/rationale:  Explain the scientific background and rationale for the investigation being reported | 2 | Background outlined in the introduction section. It focused on TB epidemiology, the physical, psychological, and financial burden of disease on the patients, and illustrated the gap in the conducted research. | Page 2-4 |
| Objectives:  State specific objectives, including any prespecified hypotheses | 3 | Objective and hypothesis clearly stated in text:  “We hypothesized that consideration of TB patients’ QoL during stages of treatment and the disease process will enable better insight into the patients’ needs, which will help in designing a tailored TB intervention program. Several studies have been performed worldwide on patients with TB infection to determine their QoL. However, literature is scarce on the QoL of TB patients in the EMR, particularly in Egypt.” | Page 4 |
| Methods | | |  |
| Study design:  Present key elements of study design early in the paper | 4 | Described  “Key elements of the study included in the method: Study design and sampling technique, Study participants and sample size, inclusion and exclusion criteria, data collection methods and tools, Selection of the control, Data analysis”. | Page 4-8 |
| Setting: Describe the setting, locations, and relevant dates, including periods of recruitment, exposure, follow-up, and data collection | 5 | Described in Study design and sampling technique:  “We randomly selected five units to conduct the study, which are: Mamoura chest hospital, Mamoura chest clinic, Bakos chest clinic, Amerya chest clinic, and kabary chest clinic). The study participants were recruited consecutively from study settings until reaching the required sample size.”  and in data collection methods and tools:  “Structured interviewing questionnaire that was collected from participants from November 20, 2021, till the end of June 2022” | Method section: page 5&6 |
| Participants:  (a) Give the eligibility criteria, and the sources and methods of selection of participants.  (b) For matched studies, give matching criteria and the number of controls per case | 6 | (a) Sample described.  “All adult males and females patients with TB, aged above 18 years and older, at any TB-treatment phase of treatment of TB (intensive phase, continuation phase treatment), and patients who were included in the district TB register attending the sampled facilities during the study period. We excluded patients with associated pulmonary diseases such as lung cancer, chronic obstructive pulmonary disease, and asthma. Patients with mental disabilities, deaf, and mute were excluded as well.”    Data sources are described in the section: Data collection methods and sources  (b) Matching described  “Several combinations of confounders which were thought to be of great impact were used for matching and the best results were obtained by using these 4 confounders: age groups, gender, presence of chronic disease other than TB, and residency.”  The number of cases and controls was 131 for each group. | Page 6&7 |
| Variables:  Clearly define all outcomes, exposures, predictors, potential confounders, and effect modifiers. Give diagnostic criteria, if applicable | 7 | Definitions of all variables, outcomes, and exposures are clearly defined in the text. | Page 4 |
| Data sources/ measurement:  For each variable of interest, give sources of data and details of methods of assessment (measurement). Describe comparability of assessment methods if there is more than one group | 8* | Source:  Described in text  “The questionnaire consisted of four sections; the first section was completed from the patient profile to ensure if he/she was eligible or not. Data collectors collected the following data from patients’ profile: date of symptom onset, date of diagnosis, treatment phase (intensive or continuation phase), current phase start date, (MDR vs drug sensitive (DS)). The second section was personal and demographic data including (age, sex, marital status, residence, education level, occupation, history of chronic diseases, and how the TB affected his/her life). Patients were categorized into low (scored <40.0%), middle (scored 40.0% to <70.0%), and high income (scored ≥70%). The third section included socioeconomic level related questions including (mother education, father education, mother work, fathers work, computer use, per capita income, family size, crowding index, sewage disposal, refuse disposal). The fourth section is the Arabic-validated WHOQOL-BREF instrument. The WHOQOL-BREF consists of 26 items, two items for evaluating general QoL and general health, and 24 items for assessing QoL in four domains, namely physical domain (seven items), psychological domain (six items), social relationship domain (three items), and environmental domain (eight items)..” | Page 7 |
| Bias:  Describe any efforts to address potential sources of bias | 9 | Described in methods.  “We recruited TB-free population from the same study settings to allow bias-free comparison of QoL scores between TB patients and TB- free population.” | Page 5 |
| Study size:  Explain how the study size was arrived at | 10 | Described. | Page 5 |
| Quantitative variables:  Explain how quantitative variables were handled in the analyses. If applicable, describe which groupings were chosen and why | 11 | Described, Statistical methods.  “continuous variables as means ± standard deviation (SD).” | Page 8 |
| Statistical methods:  a) Describe all statistical  methods, including those  used to control for  confounding  (*b*) Describe any methods used to examine subgroups and interactions  (*c*) Explain how missing data were addressed  (*d*) *Cross-sectional study*—If applicable, describe analytical methods taking account of sampling strategy  (*e*) Describe any sensitivity analyses | 12 | (a) Described:  “Assumptions of normal distribution were explored with the Kolmogorov-Smirnov test and visual inspections of the histograms. We used the independent t-test to investigate the differences between QoL domains The level of significance was set at P < 0.05” …. “ to reduce the bias due to confounders, a propensity score matching PSM was done using data extracted from a TB-free population recruited from the same study settings to allow bias-free comparison between HRQoL scores between TB patients and TB- free population.”    (b) Main stratification according to age groups and risk factors.  (c) N/A  (d) We used the independent t-test to investigate the differences between QoL domains  (e) N/A | Page 8 |
| **Results** | | | |
| Participants  (a) Report numbers of individuals at each stage of study—eg numbers potentially eligible, examined for eligibility, confirmed eligible, included in the study, completing follow-up, and analysed  (b) N/A. The outcome and the main exposure SMD were available for  all participants.  (c) Consider use of a flow diagram | 13 | (a) Described.  (b) N/A.  (c) N/A. | Page 8-10 |
| Descriptive data:  (a) Give characteristics of  study participants (e.g.  demographic, clinical,  social) and information on  exposures and potential  confounders  (b) Indicate the number of  participants with missing  data for each variable of  interest | 14 | (a) Described.  (b) N/A | Page 8-10 |
| Outcome data:  Report numbers in each  exposure category, or  summary measures of  exposure | 15 | Outcome data presented in text and tables 1 and 2 | Page 8-10 |
| Main results:  (a) Give unadjusted estimates and, if applicable, confounder adjusted estimates and their precision (eg, 95% confidence interval). Make clear which confounders were adjusted for and why they were included.  (b) Report category boundaries when continuous variables were categorized  (c) If relevant, consider  translating estimates of  relative risk into absolute  risk for a meaningful time  period | 16 | (a) Added in table 1-4  (b) Added in table 1-4  (c) N/A | Page 8-16 |
| Other analysis:  Report other analyses  done-e.g. analyses of  subgroups and interactions, and sensitivity  analyses | 17 | Main stratification according to age groups and risk factors. Table 4 | Page 15 |
| **Discussion** | | | |
| Key results:  Summarize key results with  reference to study  objectives | 18 | Done | Page 16 |
| Limitations:  Discuss limitations of the  The study, taking into account  sources of potential bias or  imprecision. Discuss both direction and magnitude of  any potential bias | 19 | Limitations were discussed in terms of conducting the study in one government, non-matching age in PSM, non-random sampling technique, recall bias, and recruiting only the adult population. | Page 19-20 |
| Interpretation:  Give a cautious overall interpretation of results considering objectives, limitations, multiplicity of analyses, results from similar studies, and other relevant evidence. | 20 | Done | Page 16-19 |
| Generalisability:  Discuss the generalizability  (external validity) of the  study results | 21 | Done  Added in the discussion section | Page 18-19 |
| Funding:  Give the source of funding and the role of the funders for the present study and, if applicable, for the original study on which the present article is based | 22 | Funding sources are given: This work was supported by a small grant from the Royal Society of Tropical Medicine and Hygiene.  Conflict of interest statement for all authors included in the manuscript. | Page 20 |

**S2 : Checking balance after PSM based on these 4 confounders**

| Participant’s characteristics | Unmatched groups comparison | | | Matched groups comparison | | |
| --- | --- | --- | --- | --- | --- | --- |
|  | Normal population  N=180 | TB patients  N=180 | P value | Normal population  N=131 | TB patients  N=131 | P value |
| Age group   - 18-30 - 31-40 - 41-50 - 51-60 - 61-75 | 52(50)  62(52.5)  43(53.8)  17(41.5)  6(35.3) | 52(50)  56(47.5)  37(46.3)  24(58.5)  11(64.7) | 0.49 | 62(76.5)  34(41.0)  16(30.2)  12(42.9)  7(41.2) | 19 (23.5)  49(59.0)  37(69.8)  16(57.1)  10(58.8) | 0.0001* |
| Gender   - Male - female | 45(25.1)  135(74.6) | 134(74.9)  46(25.4) | 0.001 | 85(50)  46(50) | 85(50)  46(50) | 1 |
| Presence of chronic diseases   - Present - Absent | 57(47.1)  123(51.5) | 64 (52.9)  116(48.5) | 0.44 | 38(45.2)  93(52.2) | 46(54.8)  85(47.8) | 0.29 |
| Residency   - Urban - Rural | 164(52.2)  16(34.8) | 150 (47.8)  30(65.2) | 0.03* | 112(51.1)  19(44.2) | 107(48.9)  24(55.8) | 0.404 |
| *: P<0.05 statistically significant | | | | | | |


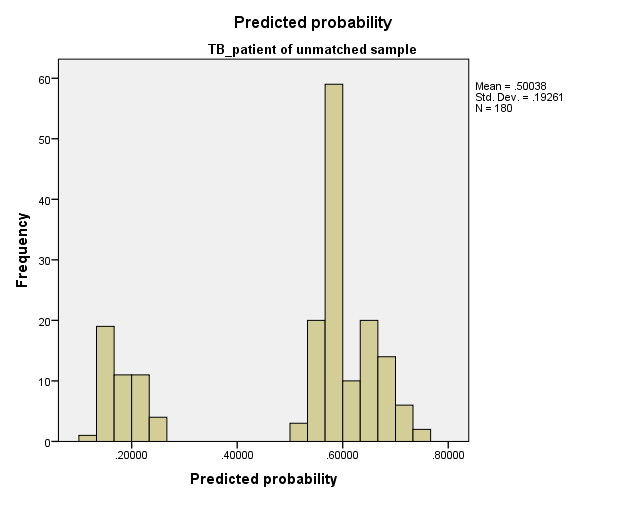

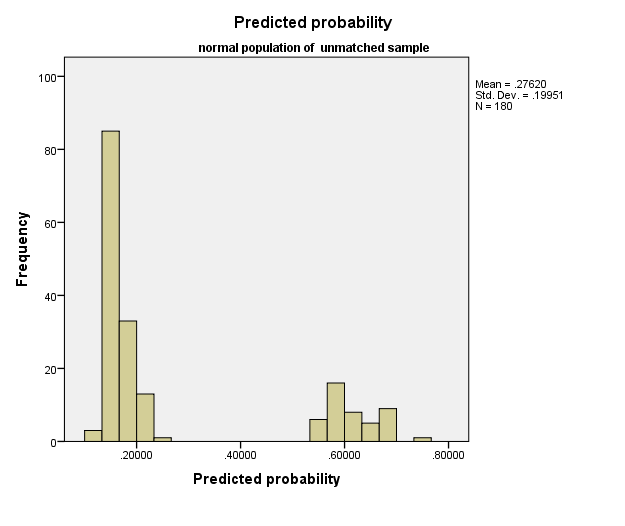


**Graph 3a Graph 3b**

**Graph (3a,3b): propensity scores distribution for TB patients and normal population of unmatched groups**

**
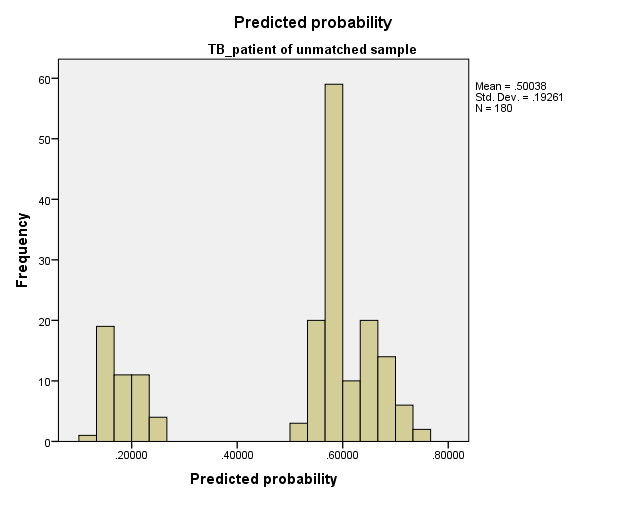

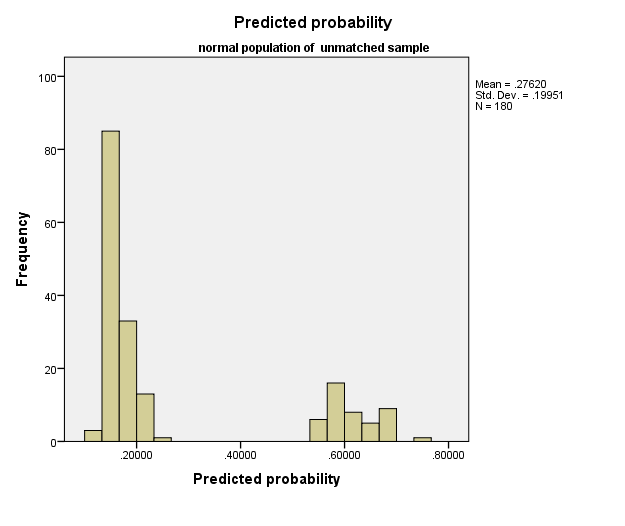
**

**Graph 4a Graph 4b**

**Graph (4a,4b): propensity scores distribution for TB- patients and normal population of matched groups**
